# Effect of Dysglycemia on Urinary Lipid Mediator Profiles in Persons With Pulmonary Tuberculosis

**DOI:** 10.3389/fimmu.2022.919802

**Published:** 2022-07-08

**Authors:** María B. Arriaga, Farina Karim, Artur T.L. Queiroz, Mariana Araújo-Pereira, Beatriz Barreto-Duarte, Caio Sales, Mahomed-Yunus S. Moosa, Matilda Mazibuko, Ginger L. Milne, Fernanda Maruri, Carlos Henrique Serezani, John R. Koethe, Marina C. Figueiredo, Afrânio L. Kritski, Marcelo Cordeiro-Santos, Valeria C. Rolla, Timothy R. Sterling, Alasdair Leslie, Bruno B. Andrade

**Affiliations:** ^1^ Laboratório de Inflamação e Biomarcadores, Instituto Gonçalo Moniz, Fundação Oswaldo Cruz, Salvador, Brazil; ^2^ Multinational Organization Network Sponsoring Translational and Epidemiological Research (MONSTER) Initiative, Salvador, Brazil; ^3^ Faculdade de Medicina, Universidade Federal da Bahia, Salvador, Brazil; ^4^ Instituto de Medicina Tropical Alexander von Humboldt, Universidad Peruana Cayetano Heredia, Lima, Peru; ^5^ Department of Infectious Diseases, Nelson R. Mandela School of Clinical Medicine, University of KwaZulu-Natal, Durban, South Africa; ^6^ Center of Data and Knowledge Integration for Health (CIDACS), Instituto Gonçalo Moniz, Fundação Oswaldo Cruz, Salvador, Brazil; ^7^ Curso de Medicina, Universidade Salvador (UNIFACS), Salvador, Brazil; ^8^ Africa Health Research Institute, Durban, South Africa; ^9^ Division of Clinical Pharmacology, Department of Medicine, Vanderbilt University School of Medicine, Nashville, TN, United States; ^10^ Division of Infectious Diseases, Department of Medicine, Vanderbilt University School of Medicine, Nashville, TN, United States; ^11^ Programa Acadêmico de Tuberculose da Faculdade de Medicina, Universidade Federal do Rio de Janeiro, Rio de Janeiro, Brazil; ^12^ Fundação Medicina Tropical Dr Heitor Vieira Dourado, Manaus, Brazil; ^13^ Programa de Pós-Graduação em Medicina Tropical, Universidade do Estado do Amazonas, Manaus, Brazil; ^14^ Universidade Federal do Amazonas, Manaus, Brazil; ^15^ Laboratório de Pesquisa Clínica em Micobacteriose, Instituto Nacional de Infectologia Evandro Chagas, Fiocruz, Rio de Janeiro, Brazil; ^16^ Division of Infection and Immunity, University College London, London, United Kingdom; ^17^ Curso de Medicina, Escola Bahiana de Medicina e Saúde Pública (EBMSP), Salvador, Brazil

**Keywords:** dysglycemia, *Mycobacterium tuberculosis*, urinary eicosanoids, lipid mediators, anti-tuberculosis treatment

## Abstract

**Background:**

Oxidized lipid mediators such as eicosanoids play a central role in the inflammatory response associated with tuberculosis (TB) pathogenesis. Diabetes mellitus (DM) leads to marked changes in lipid mediators in persons with TB. However, the associations between diabetes-related changes in lipid mediators and clearance of *M. tuberculosis* (Mtb) among persons on anti-TB treatment (ATT) are unknown. Quantification of urinary eicosanoid metabolites can provide insights into the circulating lipid mediators involved in Mtb immune responses.

**Methods:**

We conducted a multi-site prospective observational study among adults with drug-sensitive pulmonary TB and controls without active TB; both groups had sub-groups with or without dysglycemia at baseline. Participants were enrolled from RePORT-Brazil (Salvador site) and RePORT-South Africa (Durban site) and stratified according to TB status and baseline glycated hemoglobin levels: a) TB-dysglycemia (n=69); b) TB-normoglycemia (n=64); c) non-TB/dysglycemia (n=31); d) non-TB/non-dysglycemia (n=29). We evaluated the following urinary eicosanoid metabolites: 11α-hydroxy-9,15-dioxo-2,3,4,5-tetranor-prostane-1,20-dioic acid (major urinary metabolite of prostaglandin E2, PGE-M), tetranor-PGE_1_ (metabolite of PGE2, TN-E), 9α-hydroxy-11,15-dioxo-2,3,4,5-tetranor-prostane-1,20-dioic acid (metabolite of PGD2, PGD-M), 11-dehydro-thromboxane B2 (11dTxB2), 2,3-dinor-6-keto-PGF_1_α (prostaglandin I metabolite, PGI-M), and leukotriene E4 (LTE_4_). Comparisons between the study groups were performed at three time points: before ATT and 2 and 6 months after initiating therapy.

**Results:**

PGE-M and LTE_4_ values were consistently higher at all three time-points in the TB-dysglycemia group compared to the other groups (p<0.001). In addition, there was a significant decrease in PGI-M and LTE_4_ levels from baseline to month 6 in the TB-dysglycemia and TB-normoglycemia groups. Finally, TB-dysglycemia was independently associated with increased concentrations of PGD-M, PGI-M, and LTE_4_ at baseline in a multivariable model adjusting for age, sex, BMI, and study site. These associations were not affected by HIV status.

**Conclusion:**

The urinary eicosanoid metabolite profile was associated with TB-dysglycemia before and during ATT. These observations can help identify the mechanisms involved in the pathogenesis of TB-dysglycemia, and potential biomarkers of TB treatment outcomes, including among persons with dysglycemia.

## Introduction

Tuberculosis (TB) remains a major global health problem, with approximately 10 million cases worldwide in 2020 (1). Adequate TB control requires a better understanding of interactions with potential co-morbid risk factors that increase the risk of TB and poor TB treatment outcomes, including the major contributors HIV infection, undernutrition, smoking, and diabetes mellitus (DM) (2–4).

Globally, 15% of active TB cases are estimated to be attributable to DM (5); DM is associated with an increased risk of TB disease (6). We recently reported that DM in persons with TB disease increased the odds of *M. tuberculosis* (Mtb) transmission to close contacts (7). These findings highlight that control of DM (diabetes prevention and glycemic control) is important to reduce the TB burden. Furthermore, DM also increases the risk of poor TB treatment outcomes (8). The etiology of the increased risk of active TB in DM is likely multifactorial, and studies, including several from our group, have identified specific cytokine abnormalities in DM and pre-DM (both conditions are referred to hereafter as dysglycemia); dysglycemia is associated with Mtb and the development TB disease (9–12).

Understanding the effects of dysglycemia on the host immune response in the context of anti-TB therapy (ATT) could provide areas for focused, enhanced clinical management to improve outcomes. There is emerging evidence that oxidized lipid mediators play an important role in TB and DM pathogenesis (13). For instance, in Mtb infection, prostaglandin E2 (PGE_2_) inhibits necrotic cell death in macrophages, which promotes resistance to infection and host protection, while the balance between leukotriene B_4_ (LTB_4_) and lipoxin A_4_ (LXA_4_) regulates a specific level of TNFα, processes that are important for Mtb infection control (13, 14). Eicosanoid signaling pathways contribute to organ-specific insulin resistance and may also have a central role in modulating macrophage and T cell responses (15, 16). Previous studies have shown an increase in urinary 11-dTXB_2_ excretion in obese women with chronic metabolic disease (17).

Associations between the eicosanoid balance and TB clinical outcomes were demonstrated in patients with pulmonary TB but without DM in an exploratory study from our group, performed in India and China (14). We have described unique correlation profiles of plasma concentrations of pro-inflammatory lipid mediators [PGE_2_, leukotriene E_4_ (LTE_4_), and prostaglandin D_2_ (PGD_2_), among others] in individuals with TB-DM who were TB treatment naïve, delineating a molecular balance that potentially defines this comorbidity (18). More recently, plasma levels of LXA_4_, 15-epi-LxA_4_, and PGE_2_ were significantly increased, while levels of LTB_4_ were substantially decreased, in treatment-naïve persons with drug-sensitive TB-DM or TB compared to those with DM without TB or normoglycemia from South India (19). The study also demonstrated that increased levels of LXA_4_, 15-epi-LXA_4_, and the ratios of LXA_4_ to LTB_4_ and 15-epiLXA_4_ to LTB_4_ were associated with the bilateral or cavitary TB disease; concentrations of these markers were positively correlated with the Mtb burden. Importantly, after anti-TB therapy, levels of LXA_4_, 15-epi-LXA_4_, and PGE_2_ in TB-DM and TB were lower, demonstrating that the lipid mediator profile was affected by successful TB treatment (19).

These experimental and clinical studies demonstrate the importance of the eicosanoid balance and its association with pathological inflammation in both TB and DM. In addition, assessment of eicosanoid concentrations in other sample types, such as urine, may be of relevance because such samples can be easily collected and provide a clear picture of circulating lipid mediators involved in immune responses, including longitudinally on treatment. Conversely, eicosanoid levels in the blood are difficult to quantify due to low circulating levels, rapid hepatic and renal clearance, and induction of biosynthesis during sampling (20, 21).

In the present study, in a multinational cohort (Brazil and South Africa), we characterized the longitudinal urinary eicosanoid profile of TB-dysglycemia patients during anti-TB therapy and compared it with the profile of patients without dysglycemia and/or TB, also accounting for HIV infection status.

## Methods

### Ethics Statement

The study was conducted according to the principles of the Declaration of Helsinki. We used two cohorts within the Regional Prospective Observational Research in Tuberculosis (RePORT) network—one in Salvador, Brazil and one in Durban, South Africa. The study protocol was approved by the institutional review boards of the Instituto Gonçalo Moniz, Fundação Oswaldo Cruz (Brazil) and Vanderbilt University Medical Center (VUMC; United States), and the Biomedical Research Ethics Committee of the University of Kwa-Zulu Natal (South Africa). Participation in RePORT-Brazil and RePORT-South Africa was voluntary, and written informed consent was obtained from all participants.

### Study Design

This was a multinational prospective observational study with data from Brazil and South Africa. Study data were collected between June 2015 and June 2019 from the RePORT-Brazil (Salvador) site, and between December 2016 and December 2019 from the RePORT-South Africa (Durban) site.

All participants were at least 18 years old. Enrolled TB cases were evaluated at three in-person visits: (i) anti-TB treatment initiation (baseline), (ii) two months after initiating TB treatment, and (iii) after completing anti-TB treatment (month 6). Controls were selected from close TB contacts who agreed to participate in the study and were evaluated at two visits: baseline and 6 months after enrollment. In addition, telephone follow-up was performed for all participants every 6 months until 24 months from enrollment. Sociodemographic, clinical and epidemiological data such as age, sex, race/ethnicity (self-reported), and body mass index (BMI) were collected. HbA1C was measured in all participants at enrollment.

Dysglycemia was defined according to baseline HbA1c, following American Diabetes Association (ADA) guidelines (22). Individuals were classified as having DM (HbA1c≥6.5%), prediabetes (PDM; HbA1c=5.7-6.4%) or normoglycemia (HbA1c<5.7%). In this study HbA1c≥5.7% was classified as dysglycemia.

### Study Groups

#### TB Cases

In both Brazil and South Africa, we evaluated dysglycemic and normoglycemic patients with culture-confirmed drug-sensitive pulmonary TB at baseline (ATT initiation), month 2 (two months after ATT initiation [end of the intensive phase of ATT]), and month 6-9 (after completion of ATT). Clinical data such as the presence of TB symptoms (cough, fever, weight loss, fatigue, night sweats, chest pain) were obtained, and the following tests were performed: chest X-ray, HIV serologic test (only in patients without a previous diagnosis of HIV), sputum smear microscopy, Xpert-MTB/RIF (if available) and mycobacterial culture (Löwenstein-Jensen medium or BD BACTEC MGIT 960).

#### Controls

At both study sites, we also screened a cohort of TB close contacts who were normoglycemic who did not have TB. We selected who tested negative (at the baseline and six months after enrollment) for infection with Mtb by QuantiFERON-TB Gold.

### Sample Collection, Processing, and Analysis of Eicosanoid Metabolites

Concentrations of PGE-M, TN-E, PGD-M, 11dTxB_2_, PGI-M, and LTE_4_ were measured in urine at each time point in all study participants; these assays were performed at the Eicosanoid Core Laboratory at Vanderbilt University Medical Center.

[^2^H_6_]-PGE-M and [^2^H_11_]-TN-E were synthesized as described above (23, 24). [^2^H_4_]-PGI-M and [^2^H_4_]-11dTxB_2_ were purchased from Cayman Chemicals (Ann Arbor, MI USA). [20,20,20-^2^H_3_]-LTE_4_ was purchased from Enzo Life Sciences (Farmingdale, NY USA). Sep-Pak C18 and Oasis HLB (3cc/60mg) extraction cartridges were obtained from Waters Corporation (Milford, MA USA). All organic reagents were of high-performance Liquid Chromatograph (LC) quality and purchased from Sigma Aldrich (St. Louis, MO USA).

#### Prostaglandin Urinary Metabolites

After thawing, internal standards (3ng) were added to urine samples (0.5mL), and the sample was treated with methyloxime HCl to stabilize PGE-M, TN-E, PGD-M, and PGI-M. Following derivatization, the analytes were extracted using a C-18 Sep-Pak (Waters Corp. Milford, MA USA) and eluted with ethyl acetate. The samples were dried under a stream of dry nitrogen and then reconstituted in 75μL mobile phase A for ultra-pressure liquid chromatography/electrospray ionization tandem mass spectrometry (UPLC/ESI-MS/MS) analysis. LC was performed on a 2.0 x 50 mm, 1.7μm particle Acquity BEH C18 column (Waters Corporation, Milford, MA, USA) using a Waters Acquity I-Class UPLC. Mobile phase A was 95/5/0.01 (v/v/v) water/acetonitrile/acetic acid, and mobile phase B was 10/90/0.01 (v/v/v) water/acetonitrile/acetic acid. Samples were separated by a gradient of 85–5% of mobile phase A over 12 min at a flow rate of 0.375 mL/min prior to delivery to a Waters Xevo TQ-XS triple quadrupole mass spectrometer. Data were analyzed using MassLynx software and values were calculated in ng/mL.

#### Urinary LTE_4_

Urine (0.5mL) was mixed with 1.7mL water and 0.3mL 1% aqueous acetic acid. The internal standard (1ng) was added to each sample. The sample was extracted with an Oasis HLC solid phase extraction column (3cc/60mg). Samples were eluted with methanol (1mL). The eluate was evaporated under a continuous stream of dry nitrogen and then dissolved in 50μL methanol/water (85/15) and transferred to a vial for UPLC/ESI-MS/MS analysis. LC was performed on a 2.0 x 50 mm, 1.7μm particle Acquity BEH C18 column (Waters Corporation, Milford, MA, USA) using a Waters Acquity I-Class UPLC. Mobile phase A was 0.1% formic acid in water; mobile phase B was 0.1% formic acid in acetonitrile. Gradient elution was performed with 5% B for 1 min, a linear increase to 53% B until 9.5 min, a linear increase to 76% B until 11 min, a step to 100% B until 11.1 min, held for 1 min at 100% B, and re-equilibrated from 12.1 min to 14 min with 5% B. The flow rate was set to 0.400mL/min and the column temperature was maintained at 30°C. Analysis was performed using a Waters Xevo TQ-XS triple quadrupole mass spectrometer. Data were analyzed using MassLynx software and values were calculated in ng/mL.

#### Urinary Creatinine

Urinary creatinine levels were measured using a test kit from Enzo Life Sciences using 20mL aliquots that were diluted with 400mL. The urinary metabolite levels in each sample (reported as ng/mL) were normalized to the urinary creatinine level of the sample (reported in mg/mL). Final urinary eicosanoid metabolites were expressed as ng per mg creatinine.

Ultra-Performance Eicosanoid measurements in blood were not performed due to the complexity of quantification due to low circulating levels and rapid hepatic and renal clearance (21).

### Statistical Analysis

Gaussian distribution assessed by the Kolmogorov-Smirnov test. The median and interquartile range (IQR) were reported for continuous variables. Continuous variables were compared between groups according to TB and glycemic status using the Mann-Whitney *U* (between 2 groups) or Kruskal Wallis test (more than 2 groups). Changes in the eicosanoid values over time of ATT were examined using the Kruskal Wallis test with Dunn’s multiple comparisons, Friedman test, or Wilcoxon test. Categorical variables were reported as absolute values or relative frequencies and compared using the Fisher’s exact test (between 2 groups) or chi-square test (more than 2 groups), when appropriate.

Hierarchical cluster analyses with bootstrapping (100X bootstrap) were performed to describe the overall expression profile of eicosanoids in the study population. To understand the correlation between the urinary eicosanoid metabolites studied and the status of TB and TB-dysglycemia over time, we employed a network analysis based on Spearman correlation matrices as previously described (25). Profiles of correlations between biomarkers in different study groups and time points were examined using network analysis of the Spearman correlation matrices (with 100X bootstrap). Network densities were established by calculating the actual connection (known connection number between nodes) by potential connection (a connection number that could exist between two nodes), as described previously (25). Effect size was calculated with Cohen’s d (26)

A multivariable regression model using variables with univariate p-value ≤ 0.2 was performed to assess the odds ratio (OR) and 95% confidence intervals (CIs) of the associations with epidemiologic characteristics and TB-dysglycemia. All analyses were pre-specified. A p-value < 0.05 was considered statistically significant. Statistical analyses were performed using SPSS 25.0 (IBM statistics), Graphpad Prism 7.0 (GraphPad Software, San Diego, CA), and JMP 13.0 (SAS, Cary, NC, USA). All network figures were designed in gephi 0.82 with circular layout plug-in.

## Results

### Clinical Characteristics

All participants (n=193) were classified according to their status regarding TB and dysglycemia. There were 69 (36%) patients with TB-dysglycemia, 64 (33%) patients with only TB, 31 (16%) individuals with dysglycemia, and 29 (15%) with no TB or dysglycemia (**Table 1**). And when we categorize by DM and PDM, we observe that in the TB-dysglycemia group, 37 (53.6%) were diabetic and 32 (46.4%) prediabetic. In the dysglycemia group, 17 (54.8%) and 14 (45.2%) were DM and PDM, respectively. HIV infection was more frequent in the TB groups with dysglycemia (31.9%) and without dysglycemia (29.7%) than the two other groups, and only 40.9% and 31.6% respectively, used antiretroviral therapy (ART). There were no HIV-positive persons among non-TB/non-dysglycemia individuals (**Table 1**). In the TB-dysglycemia group, 13% reported use of hypoglycemic agents; none of the study participants with only dysglycemia did so.

**Table 1:** Characteristics of the study participants.

Characteristics	TB-dysglycemia	TB	Dysglycemia	non-TB/non-dysglycemia	p-value	d^&^
All	n=69	n=64	n=31	n=29		
**Male, n (%)**	46 (66.7)	39 (60.9)	9 (29.0)	8 (27.6)	**<0.001**	0.71
**Age, median (IQR)**	45 (32-55)	30 (25-40)	56.6 (44-65)	28.4 (22.8-38.0)	**<0.001**	
**Race, n (%)**					0.977	0.25
White	3 (4.3)	4 (6.3)	3 (9.7)	2 (6.9)		
Black	53 (76.8)	44 (68.8)	22 (71.0)	22 (75.9)		
*Pardo*	13 (18.8)	16 (25.0)	6 (19.4)	5 (17.2)		
**Use of hypoglycemic agents, n (%)** ^†^	9 (13)	0 (0.0)	0 (0.0)	0 (0.0)	NA	
**HIV infection, n (%)^#^**	22 (31.9)	19 (29.7)	2 (6.5)	0 (0.0)	**<0.001**	0.67
ART use, n (%)*	9 (40.9)	6 (31.6)	2 (6.5)	0 (0.0)	0.274	0.70
**BMI, (kg/m2), median (IQR)**	21.4 (18.5-24.4)	20.6 (18.5-22.2)	30.3 (27.8-34.7)	25.5 (20.6-29.4)	**<0.001**	0.34
**HbA1C (%), median (IQR)**	6.5 (6.0-9.6)	5.4 (5.2-5.5)	7.1 (5.9-9.4)	5.2 (4.9-5.4)	**<0.001**	0.47
**Glycemic status, n (%)**					NA	
Diabetes	37 (53.6%)	0 (0)	17 (54.8)	0 (0)		
Prediabetes	32 (46.4)	0 (0)	14 (45.2)	0 (0)		
Normoglycemia	0 (0)	64 (100)	0 (0)	29 (100)		
Positive AFB, n (%)**	30 (43.5)	30 (46.9)	0 (0.0)	0 (0.0)	0.694	0.07
Positive culture, n (%)**	31 (44.9)	35 (54.7)	0 (0.0)	0 (0.0)	0.260	0.06
Cavities on chest x-ray, n (%)**	49 (71.0)	40 (62.5)	0 (0.0)	0 (0.0)	0.297	0.15
Symptoms of TB, n (%)**						
Cough	66 (95.7)	61 (95.3)	0 (0.0)	0 (0.0)	0.925	0.07
Fever	41 (59.4)	37 (57.8)	0 (0.0)	0 (0.0)	0.850	0.48
Weight loss	61 (88.4)	49 (76.6)	0 (0.0)	0 (0.0)	0.071	**0.03**
Fatigue	50 (72.5)	44 (68.8)	1 (3.2)	0 (0.0)	0.638	0.51
Night sweats	40 (58.0)	47 (73.4)	0 (0.0)	0 (0.0)	0.060	0.63
Chest pain	41 (59.4)	46 (71.9)	0 (0.0)	0 (0.0)	0.131	0.65

Data represents no, (%) or median and interquartile range (IQR) and were compared using the Fisher’s exact test or Chi-squared (categorical variables) and Kruskal-Wallis (quantitative variables).

**^&^** Effect sizes by Cohen’s d (26).

† Hypoglycemic agents: Metformin and insulin.

No significant differences between TB dysglycemia vs. TB groups (p=0.852).

*The percentages were calculated with the number of individuals with HIV co-infection.

**Comparison of AFB, culture, cavities on chest x-ray and symptoms of TB were performed between TB-dysglycemic and TB cases. p-value in bold were statistically significant.

BMI, Body Mass Index; TB, Tuberculosis; AFB, acid-fast bacilli; ART, antiretroviral therapy; DM, diabetes; PDM, prediabetes; NA, Not applicable.

Persons in the TB-dysglycemia and TB groups were more likely to be male (p<0.001). The median age (56.6 years) and median body mass index (BMI) (30.3 kg/m2) of the dysglycemia group were significantly higher than the other groups (p<0.001). As expected, the levels of glycated hemoglobin (HbA1c) were highest in the dysglycemia (median=7.1%) and TB-dysglycemia (median=6.5%) groups (p<0.001). There were no significant differences in the presence of TB symptoms or AFB smear-positivity among persons with TB-dysglycemia vs. TB (**Table 1**). Clinical characteristics of the study participants by country are depicted in **Supplementary Table 1** and **Supplementary Figure 1**.

### TB-Dysglycemia Patients Have Higher Urinary Eicosanoid Concentrations Across Time Points

**Figure 1:**
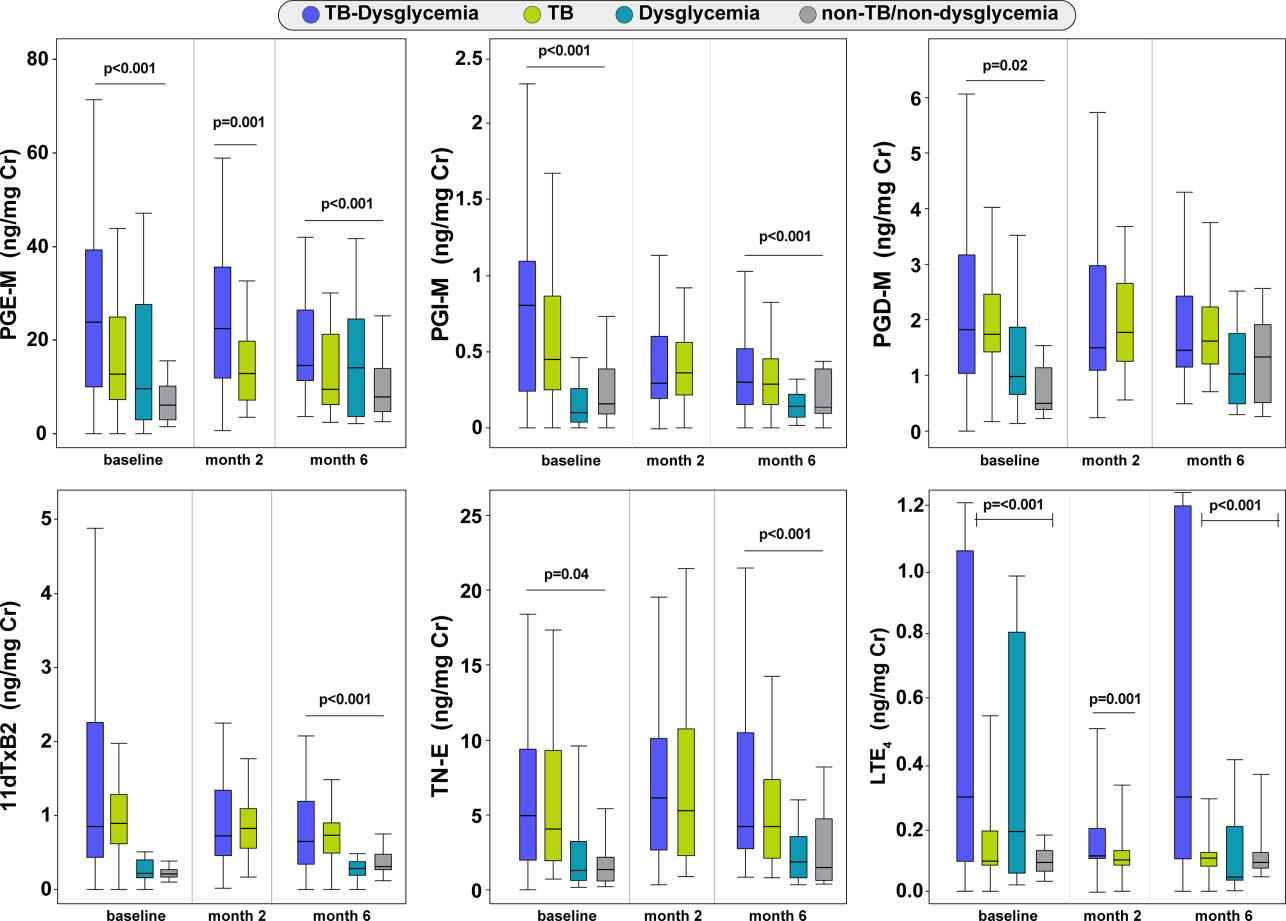
Comparison of the distribution of urinary eicosanoids between the study groups. Box plot depicting the distribution of eicosanoids (median and interquartile range) among TB-dysglycemia, TB, dysglycemic patients and non-TB/non-dysglycemia individuals at each timepoint. Groups were compared using Kruskal Wallis (for four groups) and Mann Whitney tests (for two groups). Individuals without TB (Dysglycemia and non-TB/non-dysglycemia groups) had only two visits (baseline and month 6). TB, tuberculosis; PGE-M, major urinary PGE_2_ metabolite; PGD-M, major urinary PGD_2_ metabolite; PGI-M, 2,3-dinor-6-keto-PGF_1α_ (PGI_2_ Metabolite); 11dTxB2, 11-dehydro-thromboxane B_2_ (TxB_2_ urinary metabolite); TN-E, tetranor-PGE_1_ (urinary PGE_2_ metabolite); LTE_4_, Leukotriene E_4_.

Urinary eicosanoid concentrations differed between the TB-dysglycemia, TB, dysglycemia, and non-TB/non-dysglycemia groups at several timepoints, but particularly at baseline. Interestingly, the urinary levels of PGE-M and LTE_4_ were significantly higher in the TB-dysglycemia group than in the other groups at each time point (p <0.001), while the levels of PGI-M and TN-E were also significantly higher in the TB-dysglycemia group, but only at baseline (p<0.001 and p=0.04, respectively) and month 6 (p<0.001 for both). Furthermore, PGD-M values at baseline were higher in the TB-dysglycemia group (p=0.02), as were 11dTxB2 levels at month 6 (p<0.001). (**Figure 1**). The increased urinary concentration of specific eicosanoid metabolites in the TB-dysglycemia group compared to the other groups may indicate that the inflammation mediated by these eicosanoids in these patients is greater than in patients with one or none of these conditions.

Additionally, we show urinary eicosanoid levels in each cohort (RePORT-Brazil: n=66; RePORT-South Africa n=67) by TB status and at three study time points. Urinary eicosanoid metabolite levels that significantly differed across time points in persons with TB were 11dTxB_2_ in the RePORT-Brazil cohort and PGI-M and LTE_4_ in RePORT-Brazil and South Africa cohorts. 11dTxB_2_ and PGI-M varied significantly across the timepoints in the non-TB group of RePORT-South Africa cohort is shown (**Supplementary Figure 2**).

### Dynamics of Urinary Eicosanoids Over Time in the Study Groups

**Figure 2:**
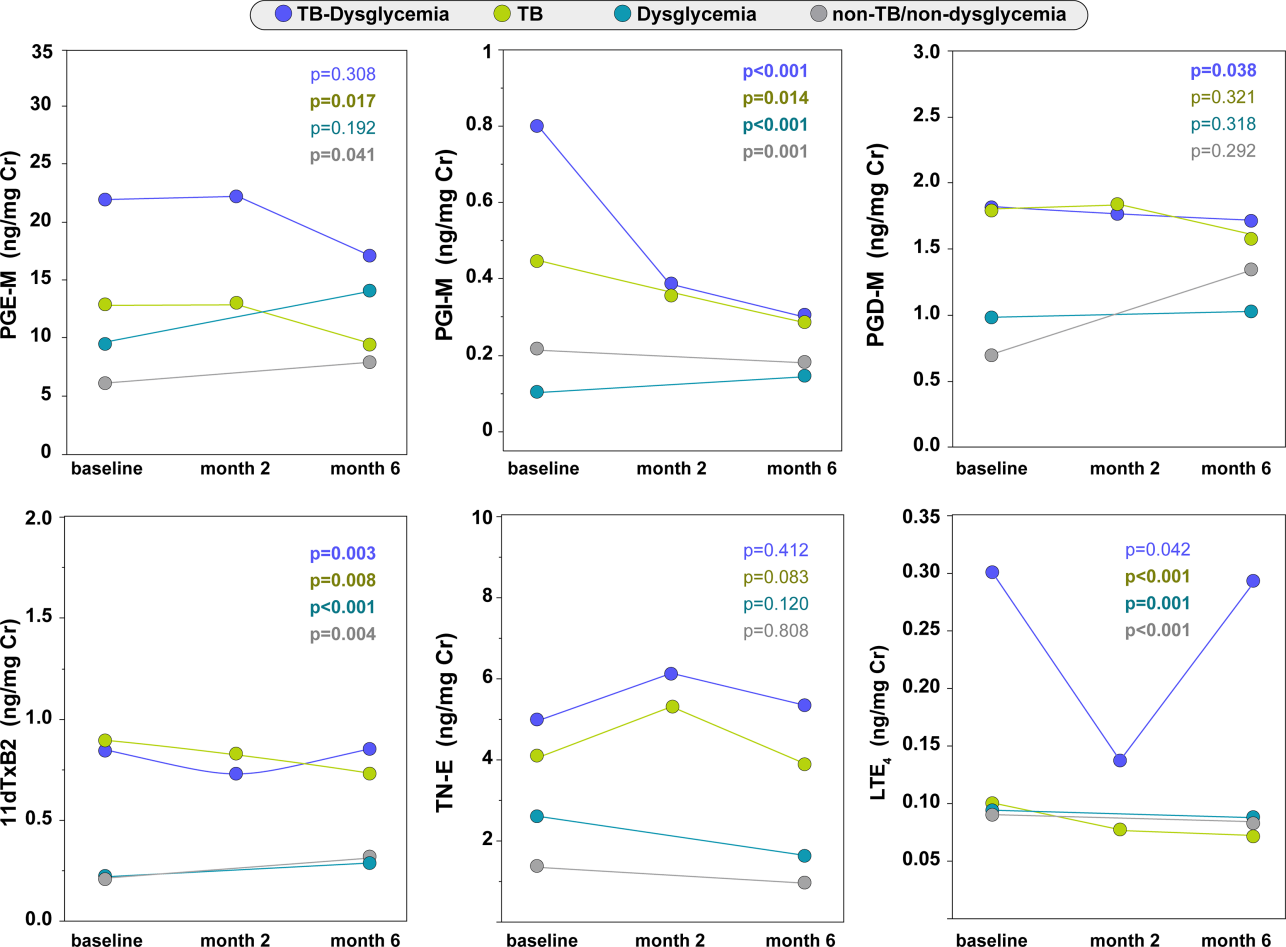
Urinary eicosanoids levels through of study timepoints. Distribution of eicosanoids (each point represents group medians) in TB-dysglycemia, TB, dysglycemia and non-TB/non-dysglycemia groups across study visits. Timepoints were compared using Friedman or Wilcoxon test (when corresponding). Individuals without TB (Dysglycemia and non-TB/non-dysglycemia groups) had only two visits (baseline and month 6). Details of central dispersion and tendency of the data are shown in the Table S2. TB, tuberculosis; PGE-M, major urinary PGE_2_ metabolite; PGD-M, major urinary PGD_2_ metabolite; PGI-M, 2,3-dinor-6-keto-PGF_1α_ (PGI_2_ Metabolite); 11dTxB2, 11-dehydro-thromboxane B_2_ (TxB_2_ urinary metabolite); TN-E, tetranor-PGE_1_ (urinary PGE_2_ metabolite); LTE_4_, Leukotriene E_4_.

We prospectively assessed urinary eicosanoid metabolite levels throughout anti-TB treatment. A trend of decrease in PGE-M was observed in both TB-dysglycemia and TB groups but only reached significance for TB only. PGE-M levels in the TB-only group remained similar between baseline and month two of treatment but then decreased at month 6 (p=0.017). However, individuals in the non-TB/non-dysglycemia group had increased PGE-M levels at month 6 compared to baseline (p=0.041) (**Figure 2** and **Supplementary Table 2**). The levels of PGI-M decreased from baseline through month 2 and month 6 in those with TB-dysglycemia (p<0.001) and those with TB (p<0.014); however, PGI-M levels increased in the dysglycemia group (p<0.001), while PGI-M levels in the non-TB/non-dysglycemia group decreased (p=0.001). PGD-M concentrations in the TB-dysglycemia group slightly decreased from baseline to month 2 and month 6 (p=0.038) (**Figure 2** and **Supplementary Table 2**). Levels of 11dTxB_2_ varied significantly in all groups through each time point. In the TB-dysglycemia group, the levels of 11dTxB_2_ had an unexpected dynamic, decreasing from baseline to month 2, then increasing from month 2 to month 6 (p=0.003). LTE_4_ values decreased from baseline to month 6 in the TB-dysglycemia (p=0.042), TB (p<0.001), and dysglycemia (p=0.001) groups but increased over this time period in the non-TB/non-dysglycemia group (p<0.001). No significant variations in TN-E levels were found in any study group (**Figure 2** and **Supplementary Table 2**).

### Correlation Between H1A1c and Urinary Lipid Mediators in TB Cases According to Dysglycemia Groups

**Figure 3:**
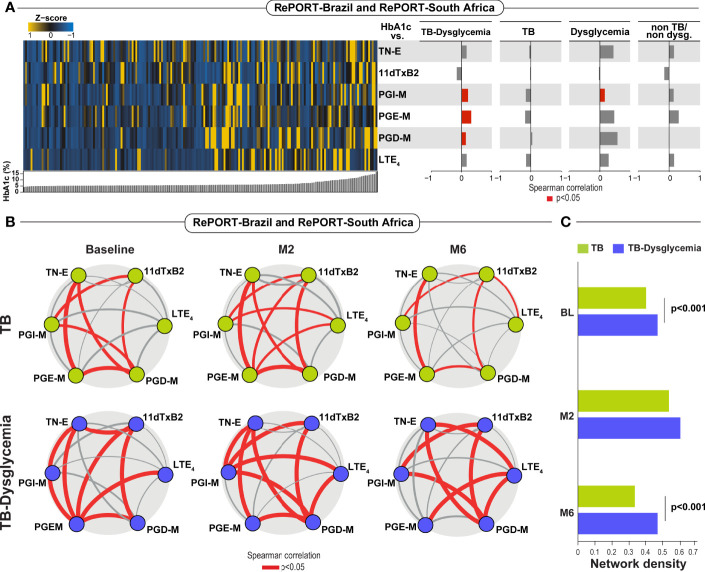
**(A)** Spearman correlation analysis plots between HbA1c (%) and TB-dysglycemia, TB, dysglycemic patients and non-TB/non-dysglycemia individuals at baseline. Significant correlations (p< 0.05) are represented in red bars. **(B)** Network analysis of eicosanoids in TB and TB-dysglycemic patients in the baseline, month 2 (M2) and month 6 (M6) among patient from Brazil and South Africa. Red solid lines: positive significant correlations (Spearman correlation). **(C)** Network densities of each bootstrap were calculated for each study group and timepoint as described in Methods. TB, tuberculosis; Dys, dysglycemia; PGE-M, major urinary PGE_2_ metabolite; PGD-M, major urinary PGD_2_ metabolite; PGIõ-M, 2,3-dinor-6-keto-PGF_1α_ (PGI_2_ Metabolite); 11dTxB2, 11-dehydro-thromboxane B_2_ (TxB_2_ urinary metabolite); TN-E, tetranor-PGE_1_ (urinary PGE_2_ metabolite); LTE_4_, Leukotriene E_4_.

Additional analyses of the eicosanoid metabolites using hierarchical clustering of Z-score normalized median data at baseline, and ordered by HbA1c values, were performed, and Spearman correlation analysis to evaluate the correlation between these parameters and HbA1c in each study group. We performed the analysis using the complete data (**Figure 3A**). We observed that the values of the eicosanoid metabolites studied were higher (yellow bars) in those patients with higher levels of HbA1c. We noted that the TB-dysglycemia group had more significant correlations than the other groups. In this group, PGI-M, PGE-M, and PGD-M had a significantly positive correlation with HbA1c levels. PGI-M was also positively correlated with HbA1c in persons with only dysglycemia (**Figure 3A**).

The network analyses between the urinary eicosanoid metabolites studied and the status of TB and TB-dysglycemia over time, revealed that TB-dysglycemia was associated with more robust and numerous correlations between urinary eicosanoid concentrations at all time points, but these correlations and network densities were statistically significant at baseline and month 6 (**Figures 3B, C**).

### TB-dysglycemia Patients With Pulmonary Cavitation Exhibit Higher Levels of Urinary PGI-M, PGD-M, and LTE_4_ Than TB Patients Without Dysglycemia

**Figure 4:**
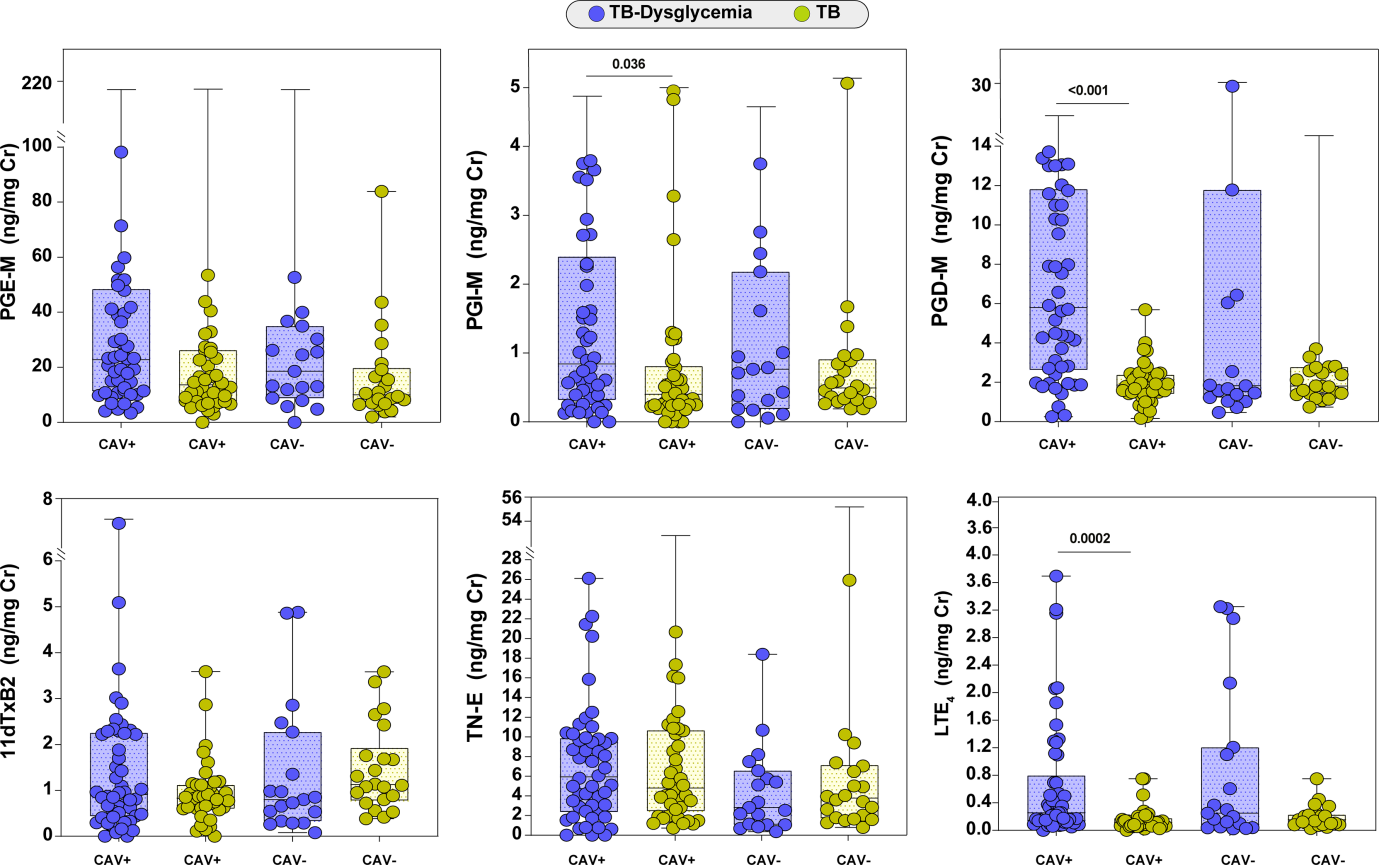
Urinary eicosanoid levels and tuberculosis severity. Scatter plot depicting the distribution of eicosanoids (median and interquartile range) among TB-Dysglycemia and TB cases with cavitation (CAV+) and without cavitation (CAV-) at baseline. Groups were compared using Kruskal Wallis test. Only statistically significant differences are shown. PGE-M, major urinary PGE_2_ metabolite; PGD-M, major urinary PGD_2_ metabolite; PGI-M, 2,3-dinor-6-keto-PGF_1α_ (PGI_2_ Metabolite); 11dTxB2, 11-dehydro-thromboxane B_2_ (TxB_2_ urinary metabolite); TN-E, tetranor-PGE_1_ (urinary PGE_2_ metabolite); LTE_4_, Leukotriene E_4_.

To evaluate the association between eicosanoid metabolite levels and disease severity in TB and TB-dysglycemic patients, we re-grouped participants with TB according to dysglycemic status and presence of pulmonary cavitation and compared urinary eicosanoid metabolite levels between groups. In **Figure 4**, we observed that levels of PGI-M (P=0.036), PGD-M (p<0.001), and LTE_4_ (p=0.0002) were higher in TB-dysglycemia participants with pulmonary cavitation than in TB group with cavitation. We did not find significant differences in urinary eicosanoid metabolite levels in TB group with cavitation and without cavitation.

### Urinary Eicosanoid Metabolites Independently Associate With TB-Dysglycemia, TB, and Dysglycemia

**Figure 5:**
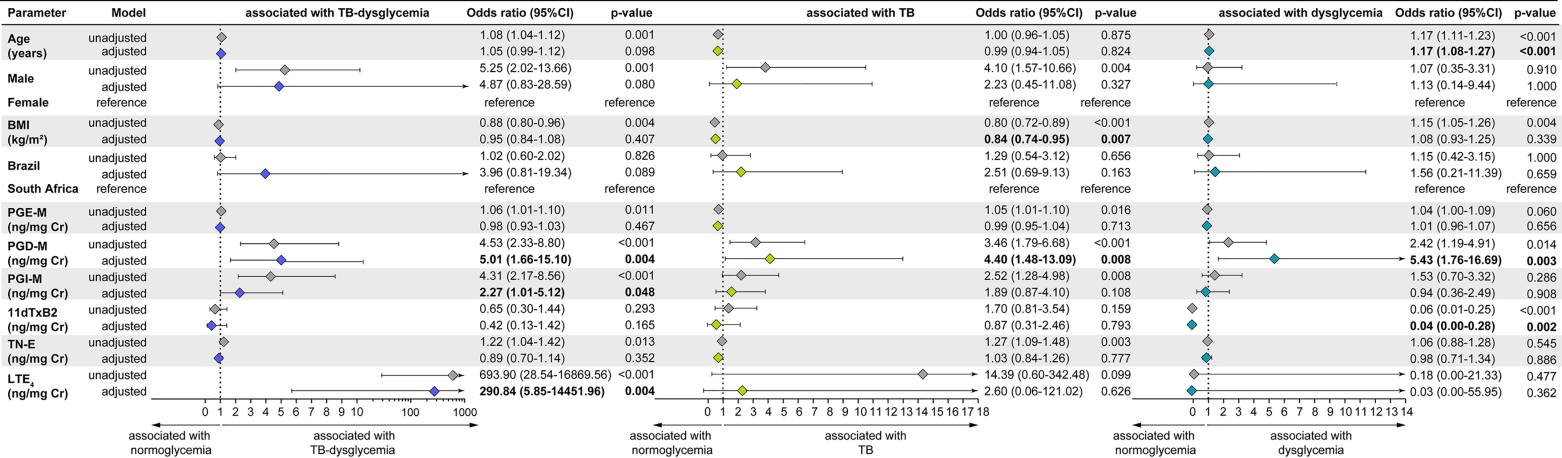
Multinomial logistic regression, adjusted for age (years), sex (male), country, PGD, PGIM, TNE, 11dTxB2, LTE4 and BMI assessed at baseline with the TB-dysglycemia condition. OR, Odds ratio; 95% CI, 95% confidence intervals; PGE-M, major urinary PGE_2_ metabolite; PGD-M, major urinary PGD_2_ metabolite; PGI-M, 2,3-dinor-6-keto-PGF_1α_ (PGI_2_ Metabolite); 11dTxB2, 11-dehydro-thromboxane B_2_ (TxB_2_ urinary metabolite); TN-E, tetranor-PGE_1_ (urinary PGE_2_ metabolite); LTE_4_, Leukotriene E_4_.

A multinomial logistic regression model adjusting for clinical factors such as age, sex, BMI, and study site revealed that increased levels of PGD-M (aOR: 5.01, 95%CI: 1.66-15.10, p=0.004), PGI-M (aOR: 2.27, 95%CI: 1.01-5.12, p=0.048) and LTE_4_ (aOR: 290.84, 95%CI: 5.85-14451.96, p=0.004) were independently associated with TB-dysglycemia (**Figure 5**). These results highlight a greater inflammatory environment by oxidized lipid mediators in patients with TB-dysglycemia.

In addition, we found that lower BMI (aOR: 0.84, 95% CI: 0.74-0.95, p=0.007) and higher PGD-M (aOR: 4.40, 95% CI: 1.48-13.09, p=0.008) were independently associated with TB (**Figure 5**). Also, higher PGD-M (aOR: 5.43, 95% CI: 1.76-16.69, p=0.003) and decreased 11dTxB_2_ (aOR: 0.04, 95% CI: 0-0.28, p = 0.002) were independently associated with dysglycemia (**Figure 5**).

Finally, we performed a logistic regression only in TB (with or without dysglycemia) patients, adjusting for clinical factors such as age, sex, BMI, study site, and HIV infection, and we found that age (aOR: 1.09, 95% CI: 1.03-1, 15, p=0.002) and increased LTE_4_ (aOR: 206.40, 95% CI: 22.46-1896.46, p<0.001) were independently associated with TB-dysglycemia. (**Supplementary Figure 3**).

## Discussion

Our study highlights the dynamics of eicosanoids in tuberculosis and ATT. TB-dysglycemia patients had higher PGE-M, TN-E, PGI-M, and LTE_4_ levels and more correlations between eicosanoid concentrations at baseline and the end of treatment. PGE-M and TN-E are two metabolites of PGE_2_; PGE_2_ has been shown to regulate different stages of the immune response during infection (27), stimulating macrophage apoptosis and heightened immunity (28). Kumar et al. (2019) also showed increased PGE_2_ in TB-DM (19). However, unlike in that study, we did not observe decreases in PGE_2_ metabolite levels during ATT.

Although the pathological interaction between dysglycemia and active TB has not been fully elucidated, recent studies have shown that patients with both diseases have a more pronounced inflammatory blood profile (29), with neutrophilic inflammation (10), a higher number of lung lesions (30) and bacterial burden (31). However, the involvement of oxidized lipid mediators in this context, and how their levels vary during anti-TB treatment, remains unclear. In the current study, we evaluated the longitudinal profile of urinary eicosanoid metabolites during ATT in a prospective multinational cohort of TB patients and for their association with TB-dysglycemia.

Our study detected a positive correlation between PGE-M and HbA1c, showing that the increase of HbA1c may be correlated with an inflammatory activation by this mediator. Additionally, metabolic inflammation, which refers to low-grade chronic systemic inflammation as opposed to classic or transient acute inflammatory responses of the innate immune system, was observed in dysglycemia and was associated with an eicosanoid balance prone towards higher leukotrienes, which are downstream products from 5-lipoxygenase enzymatic activity (32). LTE_4_ has been described as a critical proinflammatory mediator, associated with leukocyte activation, cytokine production, and trigger of macrophage necrosis (33). It is a validated biomarker of asthma disease and has been increased in diabetics (34), but little is known about this mediator in TB pathogenesis. Previous studies suggest that host-targeted therapies customized to the LTE4 genotypes may counteract the damaging effects of inadequate or excessive inflammation in patients, as shown in leukotriene inhibitors in treating asthma (35).

While roles for PGE_2_ and cysteinyl LTs in inflammation have been well-studied, PGI-M has primarily been studied for its effects on vasodilatation and inhibition of pulmonary artery smooth muscle cell proliferation (19). However, PGI-M has been described as an important inflammatory mediator in chronic diseases (36). Our results could be the basis for future studies using targeting eicosanoids such as PGI-M and LTE_4_ for host-directed therapies in individuals with TB-dysglycemia.

This study provides initial data for the research of these metabolites during and after ATT. Our data identified higher levels of both PGI-M and LTE_4_ in TB-dysglycemia patients, and a decrease in the levels of these mediators after ATT. Knowing that the pro-inflammatory profile decreases at the end of ATT (25, 37) and that the oxidized lipid mediators evaluated here may also play an inflammatory role, it is possible that PGI-M and LTE_4_ are associated with greater inflammation in patients with TB.

Notably, despite the decrease in the levels of eicosanoid metabolites after ATT, we found that the TB-dysglycemia group had a higher number and stronger correlations between the lipid mediators than patients with only TB. Also, we found that increases in PGD-M, PGI-M, and LTE_4_ were independently associated with TB-dysglycemia. This finding reinforces previous work showing that patients with TB-DM exhibit heightened plasma biomarkers of inflammation, tissue remodeling, and oxidative stress, all of which could drive increased susceptibility to adverse TB-related clinical outcomes (11).

There is evidence demonstrating deregulation of eicosanoids in immunosuppressive conditions such as HIV infection (38). However, our results showed an association between LTE_4_ and TB-dysglycemia, even after adjusting for HIV infection. We did not find previous literature that supports or refutes our results, thus more studies are needed to verify this finding.

Our cohort study consisted of patients followed at RePORT study sites in two countries with high TB prevalence: Brazil and South Africa. However, regarding the characteristics related to TB, some differences were highlighted, such as symptoms presentation, AFB positivity, and presence of cavities on chest x-ray. In both cohorts, patients with TB-dysglycemia were mainly men and had slightly higher BMI values than patients with TB and normoglycemia but much lower than patients with dysglycemia or non-TB/non-dysglycemia individuals. When comparing TB patients with and without dysglycemia, studies have shown that those normoglycemic have a lower BMI (9, 39). Even in the context of TB, this difference between BMIs can be explained by dysglycemia, given the direct association between HbA1C levels, BMI, and metabolic syndrome shown in our data has also been reported (40). Of note, in the Brazilian cohort TB-dysglycemia patients presented cavities on chest x-ray more frequently; while South African cohort TB-dysglycemia patients had symptoms such as cough, fever, weight loss, and fatigue more regularly.

This study had several limitations. First, the diabetic and prediabetic study participants at the two study sites were not homogeneous. However, the aim of our study was to evaluate dysglycemic individuals in these two settings. Second, the dysglycemia diagnosis was determined only at baseline, and a possible relationship between persistent or transient dysglycemia and eicosanoid levels during ATT was not evaluated. Third, the only method used to define dysglycemia was based on HbA1c.

With the above limitations noted, our study provides new information regarding urinary eicosanoid metabolites during ATT. We showed that TB-dysglycemia was associated with higher levels of these lipid mediators, indicating an increased inflammatory profile in patients with these co-morbidities compared to patients with only TB or dysglycemia. In addition, we also showed that PGI-M and LTE_4_ levels declined throughout treatment, indicating that treatment of TB was associated with decreased production of these mediators. Understanding the dynamics of eicosanoids in TB, especially in TB-dysglycemia, can help identify the mechanisms involved in the pathogenesis of TB-dysglycemia, and potential biomarkers of TB treatment outcomes in such patients.

## Data Availability Statement

The raw data supporting the conclusions of this article will be made available by the authors, without undue reservation.

## Ethics Statement

The studies involving human participants were reviewed and approved by Instituto Gonçalo Moniz, Fundação Oswaldo Cruz (Brazil), Vanderbilt University Medical Center (VUMC; United States), and the Biomedical Research Ethics Committee of the University of Kwa-Zulu Natal (South Africa). The patients/participants provided their written informed consent to participate in this study.

## Additional authors from the Regional Prospective Observational Research in TB (RePORT) Brazil consortium (corporate authorship)

Alice M. S. Andrade, Laboratório de Inflamação e Biomarcadores, Instituto Gonçalo Moniz, Fundação Oswaldo Cruz, Salvador, Brazil, Multinational Organization Network Sponsoring Translational and Epidemiological Research (MONSTER) Initiative, Salvador, Brazil; Michael S. Rocha, Multinational Organization Network Sponsoring Translational and Epidemiological Research (MONSTER) Initiative, Salvador, Brazil, Instituto Brasileiro para Investigação da Tuberculose, Fundação José Silveira, Salvador, Brazil; Vanessa Nascimento, Multinational Organization Network Sponsoring Translational and Epidemiological Research (MONSTER) Initiative, Salvador, Brazil, Instituto Brasileiro para Investigação da Tuberculose, Fundação José Silveira, Salvador, Brazil; Juan M. Cubillos-Angulo, Laboratório de Inflamação e Biomarcadores, Instituto Gonçalo Moniz, Fundação Oswaldo Cruz, Salvador, Brazil, Multinational Organization Network Sponsoring Translational and Epidemiological Research (MONSTER) Initiative, Salvador, Brazil, Faculdade de Medicina, Universidade Federal da Bahia, Salvador, Brazil; Hayna Malta-Santos, Laboratório de Inflamação e Biomarcadores, Instituto Gonçalo Moniz, Fundação Oswaldo Cruz, Salvador, Brazil, Faculdade de Medicina, Universidade Federal da Bahia, Salvador, Brazil; Jéssica Rebouças-Silva, Laboratório de Inflamação e Biomarcadores, Instituto Gonçalo Moniz, Fundação Oswaldo Cruz, Salvador, Brazil, Faculdade de Medicina, Universidade Federal da Bahia, Salvador, Brazil; Sayonara M. Viana Laboratório de Inflamação e Biomarcadores, Instituto Gonçalo Moniz, Fundação Oswaldo Cruz, Salvador, Brazil; Pedro Brito Laboratório de Inflamação e Biomarcadores, Instituto Gonçalo Moniz, Fundação Oswaldo Cruz, Salvador, Brazil; Saulo R. N. Santos, Instituto Brasileiro para Investigação da Tuberculose, Fundação José Silveira, Salvador, Brazil; André Ramos, Instituto Brasileiro para Investigação da Tuberculose, Fundação José Silveira, Salvador, Brazil; Alysson G. Costa, Fundação Medicina Tropical Dr Heitor Vieira Dourado, Manaus, Brazil, Programa de Pós-Graduação em Medicina Tropical, Universidade do Estado do Amazonas, Manaus, Brazil; Jaquelane Silva, Fundação Medicina Tropical Dr Heitor Vieira Dourado, Manaus, Brazil; Jamile G. de Oliveira, Secretaria Municipal de Saúde do Rio de Janeiro, Rio de Janeiro, Brazil; Aline Benjamin, Laboratório de Pesquisa Clínica em Micobacteriose, Instituto Nacional de Infectologia Evandro Chagas, Fiocruz, Rio de Janeiro, Brazil; Adriano Gomes-Silva, Laboratório de Pesquisa Clínica em Micobacteriose, Instituto Nacional de Infectologia Evandro Chagas, Fiocruz, Rio de Janeiro, Brazil; Flavia M. Sant’Anna, Laboratório de Pesquisa Clínica em Micobacteriose, Instituto Nacional de Infectologia Evandro Chagas, Fiocruz, Rio de Janeiro, Brazil; Francine P. Ignácio, Laboratório de Pesquisa Clínica em Micobacteriose, Instituto Nacional de Infectologia Evandro Chagas, Fiocruz, Rio de Janeiro, Brazil; Maria Cristina Lourenço, Bacteriology and Bioassay Laboratory, National Institute of Infectious Diseases Evandro Chagas, Oswaldo Cruz Foundation, Rio de Janeiro, Brazil; Elisangela C. Silva, Programa Acadêmico de Tuberculose da Faculdade de Medicina, Universidade Federal do Rio de Janeiro, Rio de Janeiro, Brazil; Adriana S. R. Moreira, Programa Acadêmico de Tuberculose da Faculdade de Medicina, Universidade Federal do Rio de Janeiro, Rio de Janeiro, Brazil; Mayla Mello Laboratório de Inflamação e Biomarcadores, Instituto Gonçalo Moniz, Fundação Oswaldo Cruz, Salvador, Brazil.

## Author Contributions

BA, AL, TS, CHS, and GM designed the study and mentored the work; MA, FK, AQ, MA-P, BB-D, CS, FM, MC, AK, MC-S, VR, BA, and GM performed the experiments and data collection; MA, AQ, MA-P, BB-D, CS, GM, and BA analyzed the data; MA, FK, AQ, MA-P, BB-D, CS, CHS, JK, TS, AL, and BA helped with data interpretation; MA, FK, AQ, MA-P, BB-D, TS, AL, and BA wrote the manuscript. All authors contributed to the article and approved the submitted version.

## Funding

This work was supported by the Departamento de Ciência e Tecnologia (DECIT) - Secretaria de Ciência e Tecnologia (SCTIE) – Ministério da Saúde (MS), Brazil [25029.000507/2013-07 to VR], and the National Institutes of Allergy and Infectious Diseases [U01-AI069923]. This study was also supported by the U.S. Civilian Research and Development (CRDF) (DAA3-17-63144-1, DAA3-17-63145-1, and DAA3-17-63146-1). The study was partially supported by the Intramural Research Program of the Fundaçaão Oswaldo Cruz and the Intramural Research Program of the Fundação José Silveira. The work of BA was also supported by a grant from NIH (U01AI115940). BA, AK, J.R.L.S. are senior scientists from the Conselho Nacional de Desenvolvimento Cientiífico e Tecnoloígico (CNPq). MA received a scholarship from Fundaçaão de Amparo à Pesquisa do Estado da Bahia (FAPESB). MA-P and BB-D received a research fellowship from the Coordenaçaão de Aperfeiçoamento de Pessoal de Niível Superior (CAPES, finance code: 001). AL is supported by the Wellcome Trust (210662/Z/18/Z). The funders had no role in study design, data collection and analysis, decision to publish, or preparation of the manuscript.

## Conflict of Interest

The authors declare that the research was conducted in the absence of any commercial or financial relationships that could be construed as a potential conflict of interest.

## Publisher’s Note

All claims expressed in this article are solely those of the authors and do not necessarily represent those of their affiliated organizations, or those of the publisher, the editors and the reviewers. Any product that may be evaluated in this article, or claim that may be made by its manufacturer, is not guaranteed or endorsed by the publisher.
