# Supplementary material for: Effect of Dysglycemia on Urinary Lipid Mediator Profiles in Persons With Pulmonary Tuberculosis
Source: Front Immunol. 2022 Jul 8;13:919802. doi: 10.3389/fimmu.2022.919802 (PMC9304990; doi:10.3389/fimmu.2022.919802)
Supplement: Supplementary file 1 [file DataSheet_1.docx]

Supplementary Material

# Supplementary Tables

**Supplementary Table 1. Characteristics of the study participants by country**

| Characteristics | All (n=193)  (n=193) | RePORT-Brazil  (n=96) | RePORT-SA (n=97) | p-value |
| --- | --- | --- | --- | --- |
| Male, n (%) | 102 (52.8) | 50 (52.1) | 52 (53.6) | 0.832 |
| Age, median (IQR) | 37 (27-52) | 42.5 (28.2-53.5) | 36.0 (26-49) | 0.127 |
| Race, n (%) |  |  |  | **<0.001** |
| White | 12 (6.2) | 12 (12.5) | 0 (0.0) |  |
| Black | 141 (73.1) | 44 (45.8) | 97 (100.0) |  |
| *Pardo* | 40 (20.7) | 40 (41.7) | 0 (0.0) |  |
| BMI, (kg/m2), median (IQR) | 21.9 (19.3-27.4) | 22.5 (19.9-28.3) | 21.4 (19-25.8) | 0.247 |
| HbA1C (%), median (IQR) | 5.7 (5.3-6.6) | 5.6 (5.3-7.4) | 5.7 (5.4-6.3) | 0.500 |
| Characteristics of TB patients | **(n=133)** | **(n=66)** | **(n=67)** |  |
| Dysglycemia status, n (%) |  |  |  | 0.721 |
| TB-Dysglycemia | 69 (35.8) | 31 (32.3) | 38 (39.2) |  |
| TB | 64 (33.2) | 35 (36.5) | 29 (29.9) |  |
| Dysglycemia | 29 (15) | 16 (16.7) | 15 (15.5) |  |
| Non-TB/non-dysglycemia | 31 (16.1) | 14 (14.6) | 15 (15.5) |  |
| Positive AFB^1^, n (%) | 97 (76.4) | 60 (100.0) | 37 (55.2) | **<0.001** |
| Positive culture, n (%) | 133 (100) | 66 (100.0) | 67 (100.0) | NA |
| Cavities on chest x-ray, n (%) | 92 (69.2) | 40 (41.7) | 49 (73.1) | **<0.001** |
| Symptoms of TB, n (%) |  |  |  |  |
| Cough | 127 (65.8) | 66 (100.0) | 61 (62.9) | **<0.001** |
| Fever | 78 (40.4) | 43 (65.2) | 35 (36.1) | **<0.001** |
| Weight loss | 110 (57) | 62 (93.9) | 48 (49.5) | **<0.001** |
| Fatigue | 95 (49.2) | 46 (69.7) | 49 (50.5) | **0.015** |
| Night sweats | 87 (45) | 44 (67.7) | 43 (44.3) | **0.003** |
| Chest pain | 87 (45) | 47 (71.2) | 40 (41.2) | **<0.001** |

Data represents number, (%) or median and interquartile range (IQR) and were compared using the Fisher’s exact test or Chi-squared (categorical variables) and Mann Whitney *U* test (quantitative variables). ^1^ 6 missing data in AFB test from Brazil. P-value in bold were statistically significant (<0.05). The percentages of Characteristics of TB were calculated with the total of participants.

Abbreviation: SA: South Africa; BMI: Body Mass Index TB: Tuberculosis, AFB: acid-fast bacilli, NA: Not applicable.

**Supplementary Table 2. Measurements of lipid mediators of the study participants by dysglycemia status and timepoint**

|  | **TB-dysglycemia (n=69)** | | | **TB (n=64)** | | | **Dysglycemia (n=31)** | | **non-TB/non-dysglycemia (n=29)** | |
| --- | --- | --- | --- | --- | --- | --- | --- | --- | --- | --- |
|  | **Baseline** | **Month 2** | **Month 6** | **Baseline** | **Month 2** | **Month 6** | **Baseline** | **Month 6** | **Baseline** | **Month 6** |
| **PGE-M (ng/mg Cr), median (IQR)** | 23.3 (11.5-39.9) | 22.2 (12.7-34.3) | 17.2 (12.3-26.3) | 12.8 (7.5-24.4) | 12.9 (7.3-19.5) | 11.6 (7.1-20.6) | 9.5 (3.1-24.2) | 13.8 (3.8-23.7) | 6.1 (3-10.3) | 6.9 (4.7-14) |
| **PGI-M (ng/mg Cr), median (IQR)** | 0.8 (0.3-2.3) | 0.4 (0.2-0.7) | 0.4 (0.2-0.6) | 0.4 (0.2-0.9) | 0.4 (0.2-0.6) | 0.3 (0.2-0.5) | 0.1 (0.1-0.8) | 0.1 (0.1-0.2) | 0.2 (0.1-0.3) | 0.2 (0.1-0.3) |
| **PGD-M (ng/mg Cr), median (IQR)** | 5.2 (1.9-11.7) | 1.8 (1.1-2.9) | 1.8 (1.2-2.5) | 1.8 (1.4-2.5) | 1.8 (1.3-2.7) | 1.6 (1.2-2.1) | 0.9 (0.5-1.2) | 1.4 (0.6-3) | 0.5 (0.4-0.9) | 1.5 (0.5-2) |
| **11dTxB2 (ng/mg Cr), median (IQR)** | 0.9 (0.4-2.2) | 0.7 (0.5-1.3) | 0.9 (0.5-1.2) | 0.9 (0.6-1.3) | 0.8 (0.6-1.1) | 0.7 (0.5-0.9) | 0.2 (0.2-0.4) | 0.3 (0.2-0.4) | 0.2 (0.2-0.3) | 0.3 (0.3-0.4) |
| **TN-E (ng/mg Cr), median (IQR)** | 5.1 (2.1-9.5) | 6.1 (2.8-10.1) | 4.8 (2.6-10.1) | 4.1 (2-9.2) | 5.3 (2.3-10.6) | 4.6 (2.5-8.6) | 1.4 (0.6-2.6) | 1.6 (0.9-3) | 1 (0.5-1.4) | 1.2 (0.6-4.7) |
| **LTE_4_ (ng/mg Cr), median (IQR)** | 0.3 (0.1-1.1) | 0.1 (0.1-0.2) | 0.3 (0.1-1.2) | 0.1 (0.1-0.2) | 0.1 (0-0.1) | 0.1 (0.1-0.1) | 0.2 (0.1-0.8) | 0.1 (0-0.2) | 0.1 (0.1-0.1) | 0.1 (0.1-0.1) |

Data represents median and interquartile range (IQR). Abbreviations: TB: tuberculosis, PGE-M: major urinary PGE_2_ metabolite, PGD-M: major urinary PGD_2_ metabolite, PGI-M: 2,3-dinor-6-keto-PGF_1α_ (PGI_2_ Metabolite), 11dTxB2: 11-dehydro-thromboxane B_2_ (TxB_2_ urinary metabolite), TN-E: tetranor-PGE_1_ (urinary PGE_2_ metabolite); LTE_4_: Leukotriene E_4_.

# Supplementary Figures


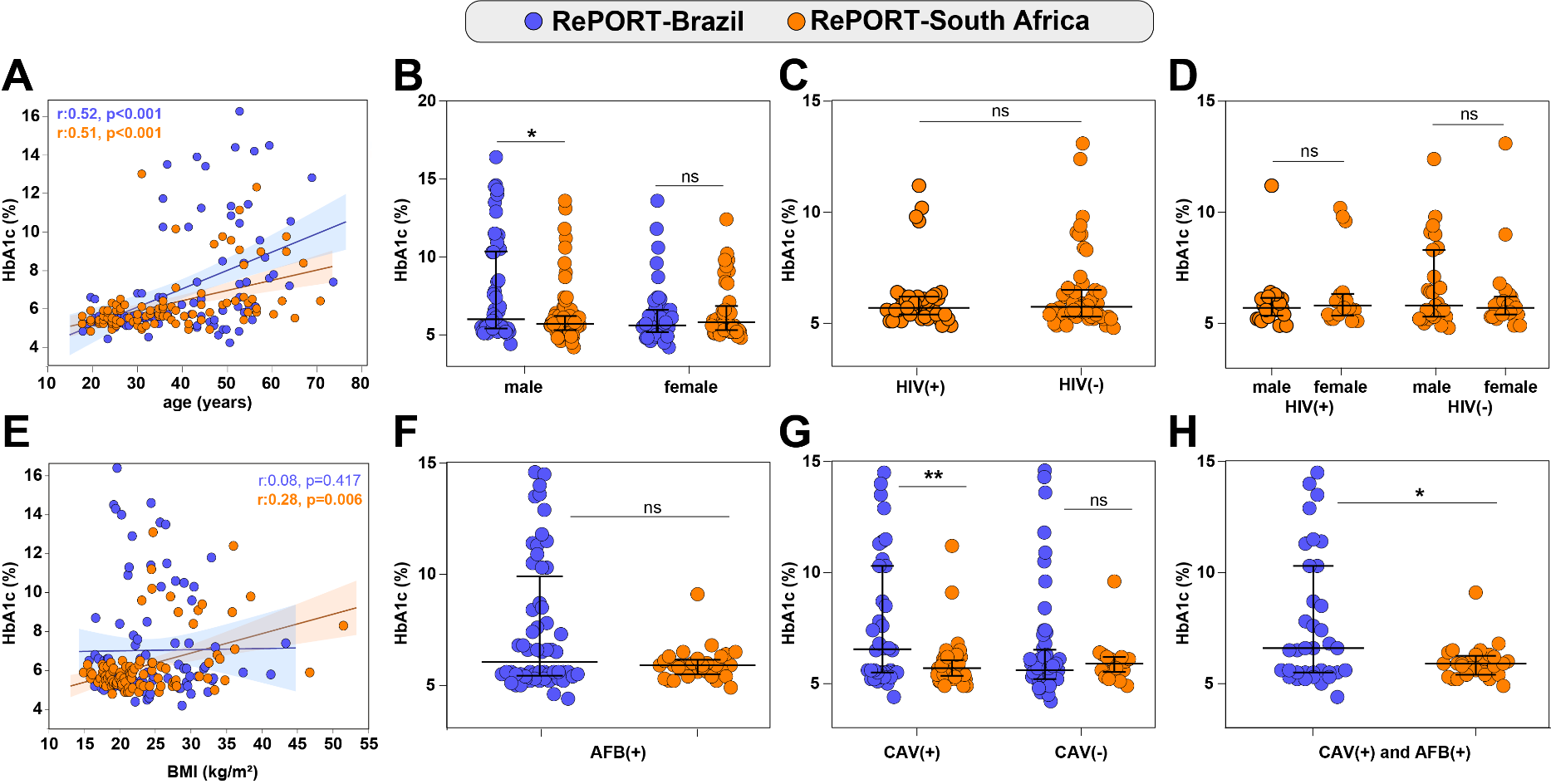


**Supplementary Figure 1. HbA1c and clinical characteristics of the study participants by country.** Spearman correlation between glycated hemoglobin (HbA1c) (%) and **(A**) age (years) and **(E)** Body Mass Index (BMI). **(B)** Distribution of HbA1c % by sex, **(C)** HIV infection, **(D)** sex and HIV infection, **(F)** Acid-Fast Bacilli (AFB) smear-positive, **(G)** pulmonary cavitation (CAV) and **(H)** pulmonary cavitation and AFB smear-positive. In blue Report-Brazil data and in orange Report-South Africa data. *p < 0.05; **p< 0.01.


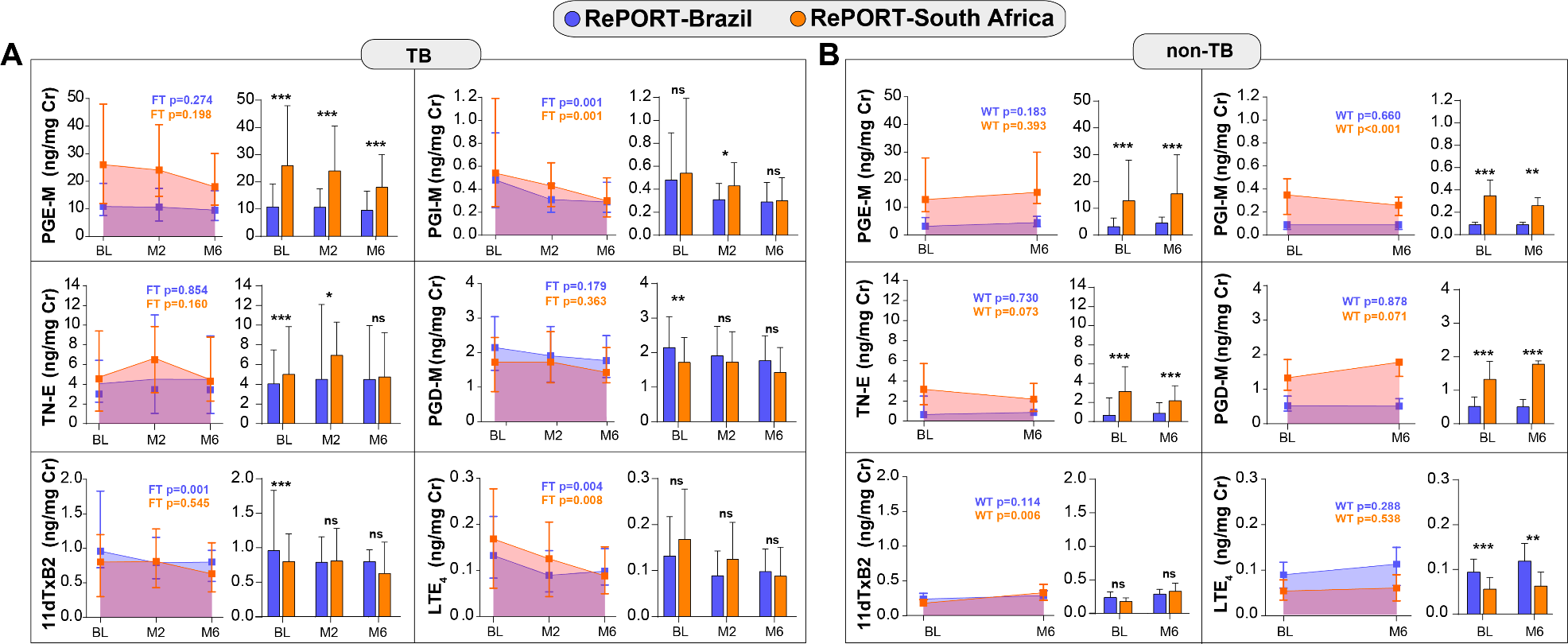


**Supplementary Figure 2. Distribution of eicosanoids in individuals from RePORT-Brazil and RePORT-South Africa cohorts by timepoints. (A)** Patients with tuberculosis (TB) and **(B)** Participants without TB. The areas were formed with the medians and interquartile ranges of the levels of each eicosanoid through each timepoints. On the side, the bars show the medians and interquartile ranges at each timepoint compared between countries. Study timepoints for each cohort were compared using Friedman (FT) and Wilcoxon test (WT), when applicable. Mann-Whitney *U* test was used for compared eicosanoids levels between cohorts in each timepoint. Individuals without TB (Dysglycemia and non-TB/non-dysglycemia groups) had only two visits (baseline and month 6). *p < 0.05; **p< 0.01; *** p<0.001.

Abbreviations: PGE-M: major urinary PGE_2_ metabolite, PGD-M: major urinary PGD_2_ metabolite, PGI-M: 2,3-dinor-6-keto-PGF_1α_ (PGI_2_ Metabolite), 11dTxB2: 11-dehydro-thromboxane B_2_ (TxB_2_ urinary metabolite), TN-E: tetranor-PGE_1_ (urinary PGE_2_ metabolite); LTE_4_: Leukotriene E_4_.


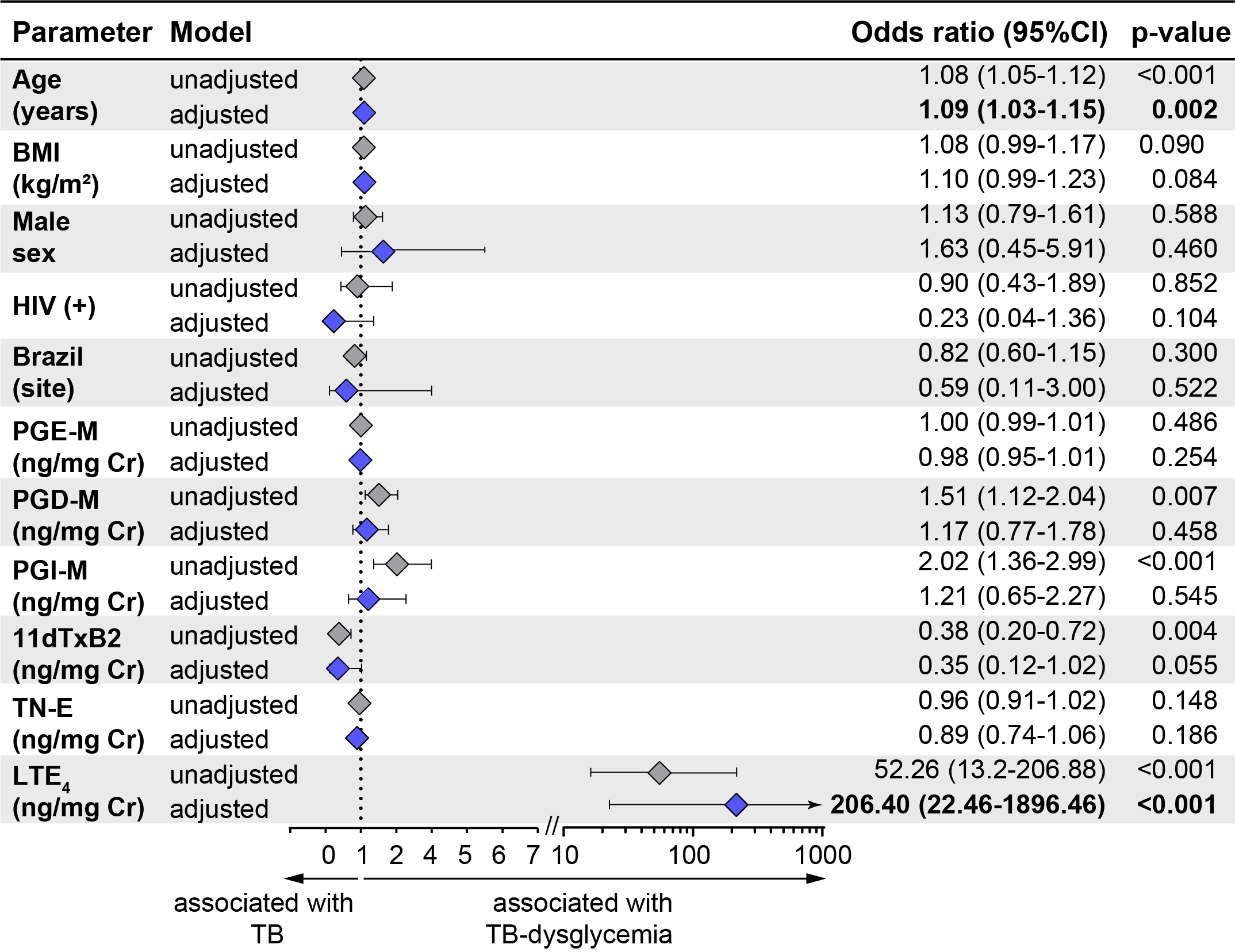


**Supplementary Figure 3.** Logistic regression, adjustment for age (years), BMI, sex (male), HIV infection, country (site), PGE-M, PGD-M, PGI-M, TN-E, 11dTxB2, and LTE_4_ assessed at baseline with the TB-dysglycemia condition.

Abbreviations: BMI: Body Mass Index, OR: Odds ratio; 95% CI: 95% confidence intervals, PGE-M: major urinary PGE_2_ metabolite, PGD-M: major urinary PGD_2_ metabolite, PGI-M: 2,3-dinor-6-keto-PGF_1a_ (PGI_2_ Metabolite), 11dTxB2: 11-dehydro-thromboxane B_2_ (TxB_2_ urinary metabolite), TN-E: tetranor-PGE_1_ (urinary PGE_2_ metabolite); LTE_4_: Leukotriene E_4_.
